# Estimation of cancer risk due to exposure to cadmium contamination in herbal products in Thailand

**Published:** 2015-01-14

**Authors:** Viroj Wiwanitkit

**Affiliations:** Surin Rajabhat University, Surin, Thailand

**Keywords:** Cancer risk, Cadmium, Herbal drugs

**Implication for health policy/practice/research/medical education:**

Contamination in herbal products becomes the big problem at present. Many dangerous chemical can be seen in many herbal products. Cadmium is a heavy metal that can be a possible contaminant.

At present, the use of alternative and complimentary medical product becomes an important concern in modern medicine. A lot of patients find and seek such products for using in disease management. Contamination in herbal products becomes the big problem at present. Many dangerous chemical can be seen in many herbal products. Cadmium is a heavy metal that can be a possible contaminant. According to a recent study by Ramezani et al, high level of cadmium can be seen in herbal plants (1). Intoxication due to intake of contaminated product is of concern. Exposure to cadmium can result in nephropathy and the chronic exposure is related to renal carcinogenesis (2,3). Focusing on kidney cancer, Liu et al noted that the cadmium-induced oxidative stress was related to the aberration of gene which further resulted in renal toxicity, renal pathology and carcinogenesis (4). Here, the authors assess cancer risk from oral intake of cadmium contaminated Thai herbal products using the standard technique previously published by Wiwanitkit (5). Individual lifetime cancer risk is calculated using the following equation, by “concentration of contaminated lead in herbal product × lifetime unit risk factor.” The referencing unit risk factor of cadmium is equal to 1.8 × 10^−3^ m^3^/μg (http://rais.ornl.gov/tox/profiles/cadmium.html). Focusing on the cadmium contamination, according to the recent study on 86 product samples collected from Bangkok Metropolitan region, the prevalence rate of cadmium contamination is equal to 24.4% (6). According to that study, the average concentration of cadmium is equal to 23.9 ppm or 23.9 × 10^3^ μg/kg (referencing cadmium density equal to 8650 kg/m (3); http://www.webelements.com/cadmium/) . According to these basic information, the derived individual lifetime cancer risk is equal to 372132. Adjusted with the prevalence of contamination, the estimated cancer risk of a person due to oral intake of herbal product is equal to 90800.2. This rate is extremely high and it seems that the use of local herbal product by local people significantly increases the chance to have a renal cancer in their lifetime.

## Author’s contribution

VW is the single author of the paper.

## Conflicts of interest

The author declared no competing interests.

## Ethical considerations

Ethical issues (including plagiarism, data fabrication, double publication) have been completely observed by the author.

## Funding/Support

None.
